# Pesticide effects of highly stable green synthesized silver nanocomposites to be used in organic tomato crops

**DOI:** 10.1038/s41598-025-03101-9

**Published:** 2025-07-01

**Authors:** Luis E. Trujillo, Pablo Landázuri, Carlos Noceda, José Velasco, Luz Toapanta, Giulliana Criollo, Andrea Poaquiza, Fulton Barros, Vladimir Aguirre, Andrés Izquierdo, Julio Chacón, Zailmar Morales, Alexis Debut

**Affiliations:** 1 Life Science and Agriculture, Universidad de las Fuerzas Armadas-ESPE, Industrial Biotechnology Research Group Sangolquı, Sangolquí, 171103 Ecuador; 2 Center for Nanoscience and Nanotechnology (CENCINAT), Universidad de las Fuerzas Armadas (ESPE), Sangolquí, 171103 Ecuador; 3 Yachay Tech.University, Urcuquí, 100115 Ecuador

**Keywords:** AgNPs, PVP, Nanocomposite, Thermogravimetry, Thermal stability, Whitefly, Fungi, Biotechnology, Microbiology, Plant sciences

## Abstract

Greenhouse whitefly, *Trialeurodes vaporariorum* Westwood (Hemiptera: Aleyrodidae) together with the negative incidence of fungi such as *Oidium neolycopersici* and phytopathogenic bacteria, are responsible for causing serious economic losses in organic tomato crops. Silver nanoparticles (AgNPs) are a promising solution to problems caused by these pests due to their insecticidal and bactericidal properties. However, these compounds are unstable and tend to form agglomerates. This fact causes them to lose their properties so, preventing its use as an alternative to chemical pesticides in organic cultures. In this research, a novel one-step green synthesis method to obtain silver stable nanocomposites using rosemary extract (*Rosmarinus officinalis* L.) as green reducing agent was stablished. The polymer polyvinylpyrrolidone (PVP) was used additionally in the same synthesis reaction as AgNPs stabilizing agent. With this scalable one step synthesis, the obtained PVP-AgNPs nanocomposite showed particle sizes of 10.8 nm being highly stable during 326 days. At different assayed doses, this highly stable PVP-AgNPs nanocomposite, was able to control whitefly specimens efficiently with an average mortality rate of 98% after 10 days of the nanocomposite application to naturally infested tomato leaves grown under greenhouse conditions. Additionally, in a diffusion inhibition assay on agar plates, inhibition of *Bacillus amyloliquefaciens*, *Pseudomonas syringae*, and *Xanthomonas* sp growth was found. PVP-AgNPs nanocomposite was also effective to control *Oidium neolycopersici* in greenhouse grown tomato plants. To our knowledge, this is the first well-founded report related to a PVP-AgNPs nanocomposite obtained by green synthesis using rosemary extracts as reducing agent able to control whitefly and tomato powdery mildew, being a potential alternative to chemical pesticides in organic tomato crops.

## Introduction

Tomato and its different species (*Solanum lycopersicum* L.; *Solanum lycopersicum* var. cerasiforme) are important crops worldwide^1^. However, around 100 insect pests and 25 non-insect pests have been documented to attack tomatoes, affecting the hold plant, fruits number and quality^2,3^. Regarding diseases, over 200 species including fungi, bacteria and viruses have been identified as tomato pathogens causing production problems^4^, especially in organic cultivation where chemicals are not used^5^. Among the main pests, the greenhouse whitefly, *Trialeurodes vaporariorum* Westwood (Hemiptera: Aleyrodidae), together with the negative incidence of fungi and phytopathogenic bacteria, are responsible for causing serious economic losses for this.

crop industry^6^.

*Trialeurodes vaporariorum* Westwood (Hemiptera: Aleyrodidae) for example, is also responsible of transmitting viruses to plants^6–9^ producing a certain molasses that favors the growth of fungi, affecting photosynthetic efficiency and therefore final yields^10,11^.On the other hand, crop losses as result of phytopathogens attacks account for about 16% of the total crop production worldwide being mainly fungi responsible for 70–80% of these losses^12,13^.

Given the incidence of these biotic factors, farmers prefer to use pesticides to control them however; although pesticides protect efficiently target pests, their indiscriminate use is associated with other consequences, such as costs increments, chemical residues in the field, pollution, human health problems and one of the most important issue related to insect resistance and.

insect pest resurgence^14^.

In recent years, new technologies such as nanotechnology have begun to be applied in modern agriculture as a promising alternative to be implemented in integrated management programs^15^. The use of nanoparticles (NPs) in general, has shown a variety of applications in agriculture, as plant growth promoters, nutrient transporters and potential pesticides^16^.

Silver nanoparticles (AgNPs) among them, have been one of the most studied nanomaterials in recent decades. AgNPs show diverse useful properties, which vary significantly depending on the used synthesis method, being mainly of agronomic interest due to their antimicrobial and insecticidal properties^17,18^. Therefore, the use of AgNPs obtained through different synthesis methods to be used as new pesticides has gained great attention.

An impressive number of studies have been carried out to test the toxic potential of these nanoparticles against a large number of pests and vectors, with special emphasis on mosquitoes, ticks and mites^19^. However, there are not many reports on the effect of highly stable AgNPs nanocomposites formulation on greenhouse whiteflies at their different stages or even silver nanocomposites antibacterial or antifungal effects mainly against *Oidium neolycopersici*.

Therefore, the objective of this research was to standardize by the first time a scalable single step biological green synthesis method using rosemary (*Rosmarinus officinalis* L.) extract as reducing agent in the presence of PVP as stabilizing polymer to obtain AgNPs nanocomposites to be used as pesticide. With this strategy, the obtained nanocomposites formulation showed highly stable nanoparticles with uniform sizes and distribution. This fact allows us to evaluate the PVP-AgNPs insecticidal and antimicrobial potential to use it later as raw material for a nanoemulsion formulation pointed to organic tomato crops protection that could be applied to other organic crops. This fact will guarantee the non-use of chemical pesticides in organic crops.

## Results and discussion

### PVP-AgNPs nanocomposite synthesis and charactherization

**Fig. 1:**
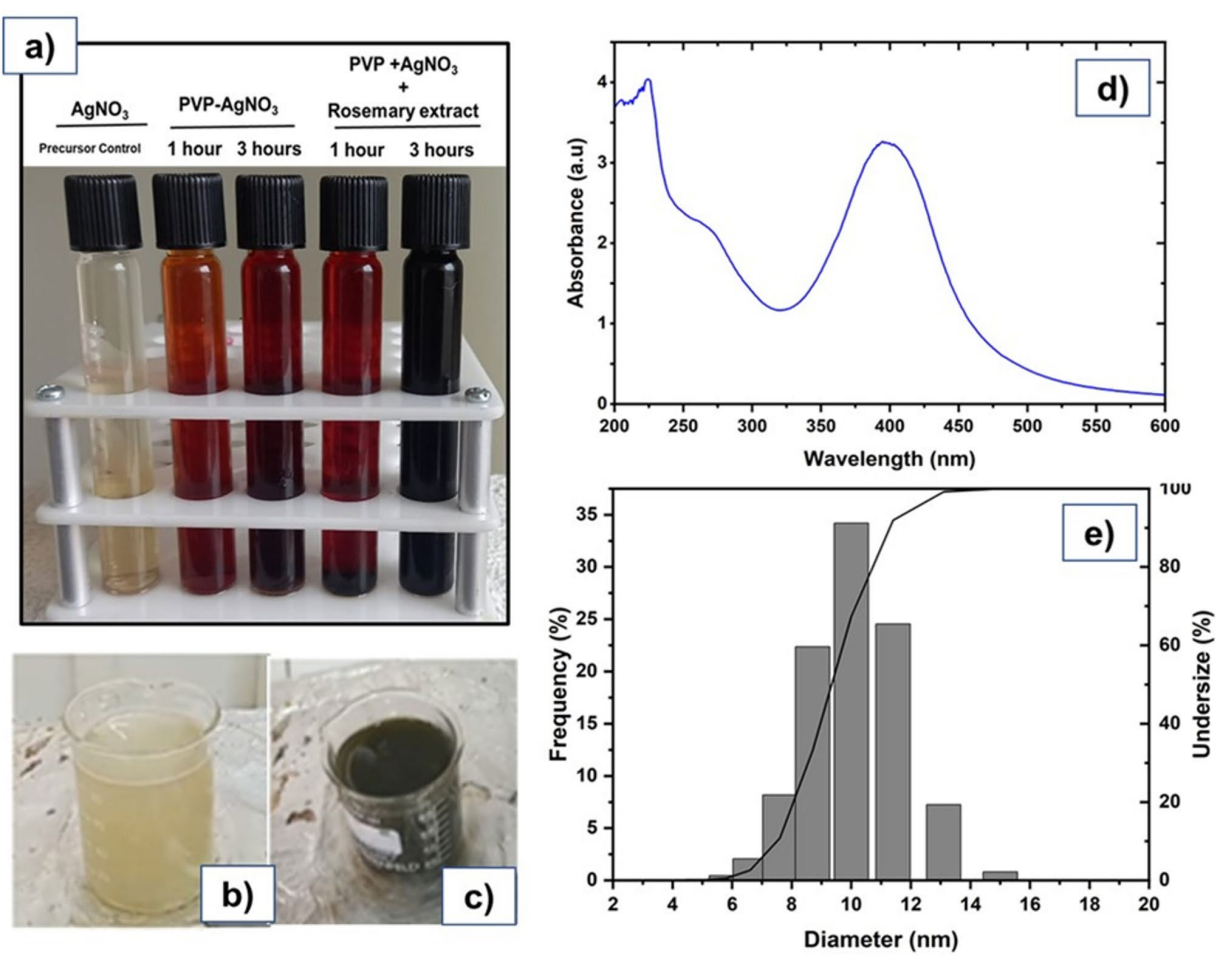
Synthesis and characterization of PVP-AgNPs nanocomposite. (**a**) Process of AgNO_3_ protection and reduction by adding PVP and rosemary extract separately at 1 and 3 h. (**b**) and (**c**) Single step of PVP-AgNPs synthesis as described in methods, showing color change from the precursor AgNO_3_ (**b**) until PVP-AgNPs formation (**c**), (**d**) UV–Vis spectrum, showing the absorbance peak of PVP-AgNPs located at 398 nm. (**e**) Size distribution histogram, with a mean hydrodynamic diameter of 10.8 nm.

Figure 1a show the color change of the precursor solution containing only sodium hydroxide and silver nitrate, from a transparent to a dark brown color tone by the separate addition of PVP and rosemary (*Rosmarinus officinalis* L.) extract at different times corresponding to 1 and 3 h. On the other hand, Fig. 1b,c display color change when both, PVP as a stabilizing agent and rosemary extract as reducing agent were added in a single synthesis reaction for 3 h as described in methos. Dark color reached after 3 h single step synthesis indicated complete reduction and so, successful nanocomposite synthesis.

Within organic agriculture, the biological synthesis method using plant extracts is the most used, since it does not require dangerous compounds affecting environment, and additionally, obtained AgNPs have different characteristics compared to other synthesis methods^20^. In addition, they exhibit low or no toxicity compared to chemical methods^21^.

The main advantage of using plant extracts including rosemary extract for AgNPs green synthesis is the vast availability of plant material in nature. Due to the wide range of secondary metabolites present in the plant extracts, efficiently help to metal reduction in a fast way^22^. Besides, flavonoids, steroids and carbohydrates provide coating activity and stability to the synthesized nanoparticle^23^. Contrary, main disadvantage of AgNPs synthesis by common chemical methods is that the synthesized nanoparticles tend to agglomerate or undergo modifications on their surface, which can influence negatively in its stability decreasing its toxicity against different organisms. Undoubtedly, this fact limits its use as an efficient pesticide. To mitigate these problems, functionalization of nanoparticles surface using biodegradable polymer coatings has been proposed^24^. This type of nanoparticles functionalization and stabilization using polymers is known as steric method^25^. In the synthesis method proposed in this research, we used Polyvinylpyrrolidone (PVP), an ecofriendly biodegradable polymer general regarded as safe (GRAS), able to stabilize and control AgNPs size and shape^26^. By itself, it is capable to reduce silver ions Ag + from a salt precursor to metallic silver Ag0 but slowly, which can result in large nanoparticles with a polydisperse size distribution.

To confirm the PVP-AgNPs formation it is necessary to carry out their characterization to evaluate their functional aspects, mainly size and concentration^27,28^. In that sense, UV-vis spectroscopy characterization showed the presence of a maximum absorbance peak of 3,256 at a wavelength of 398 nm corresponding to PVP-AgNPs as shown in Fig. 1d.

In previous reports^29,30^ researchers used PVP as stabilizing agent, and sodium borohydride as a reducing agent in a non-green synthesis procedure. They found an absorbance spectrum at wavelength between 395 and 410 nm, which indicated the presence of AgNPs between 395 and 398 nm depending on the used PVP concentration, reaching maximum absorbance peaks between 0.42 and 1.2. Furthermore, they pointed out that at a highest PVP concentration, agglomerates formation occurs due to extensive time of the synthesis method.

Interestingly, the maximum absorbance peak of 3.256 obtained in our research, was higher than peaks reported in the literature suggesting that the use of rosemary extract (*Rosmarinus officinalis* L.) and PVP at a lower concentration than that reported in previous research produces a higher nanoparticles concentration due to the synergistic effect that improves the PVP-AgNPs quality and stability.

**Fig. 2:**
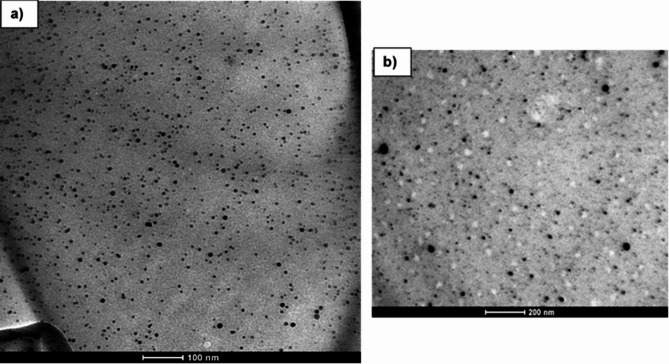
TEM images of the PVP-AgNPs nanocomposite at (**a**) 100 nm and (**b**) 200 nm, scale respectively, showing adequate PVP-AgNPs size and no particles agglomeration.

On the other hand, DLS measurements of PVP-AgNPs size and specific surface area^31^ displayed that the average size found was 10.8 nm (Fig. 1e) with a surface area of 5.778 × 10^6^ (cm^2^/cm^3^). The obtained PVP-AgNPs size was subsequently confirmed using TEM microscopy at 100, 200 nm scale showing no particles agglomeration and homogeneous adequate size as shown in Fig. 2a, b.

According to our findings, the use of PVP in the nanocomposite synthesis undoubtedly showed and reinforced the findings of previous investigations regarding the positive effects on PVP-AgNPs size due to the polymer stabilizing effect, which prevents agglomeration so, a more uniform size distribution of the PVP-AgNPs was obtained. Similar to our findings, other authors reported that the PVP polymer can be used as a coating agent, accelerating the reaction time and helping to control nucleation to form smaller metallic particles^32–34^. In this way, polymeric matrix can protect AgNPs from degradation changing their dynamic behavior^35^. Furthermore, the surface area may suggest better nanoparticles dispersion and greater interaction with the surrounding medium, reflecting the formation of nanoparticles with great stability^36^.

**Fig. 3:**
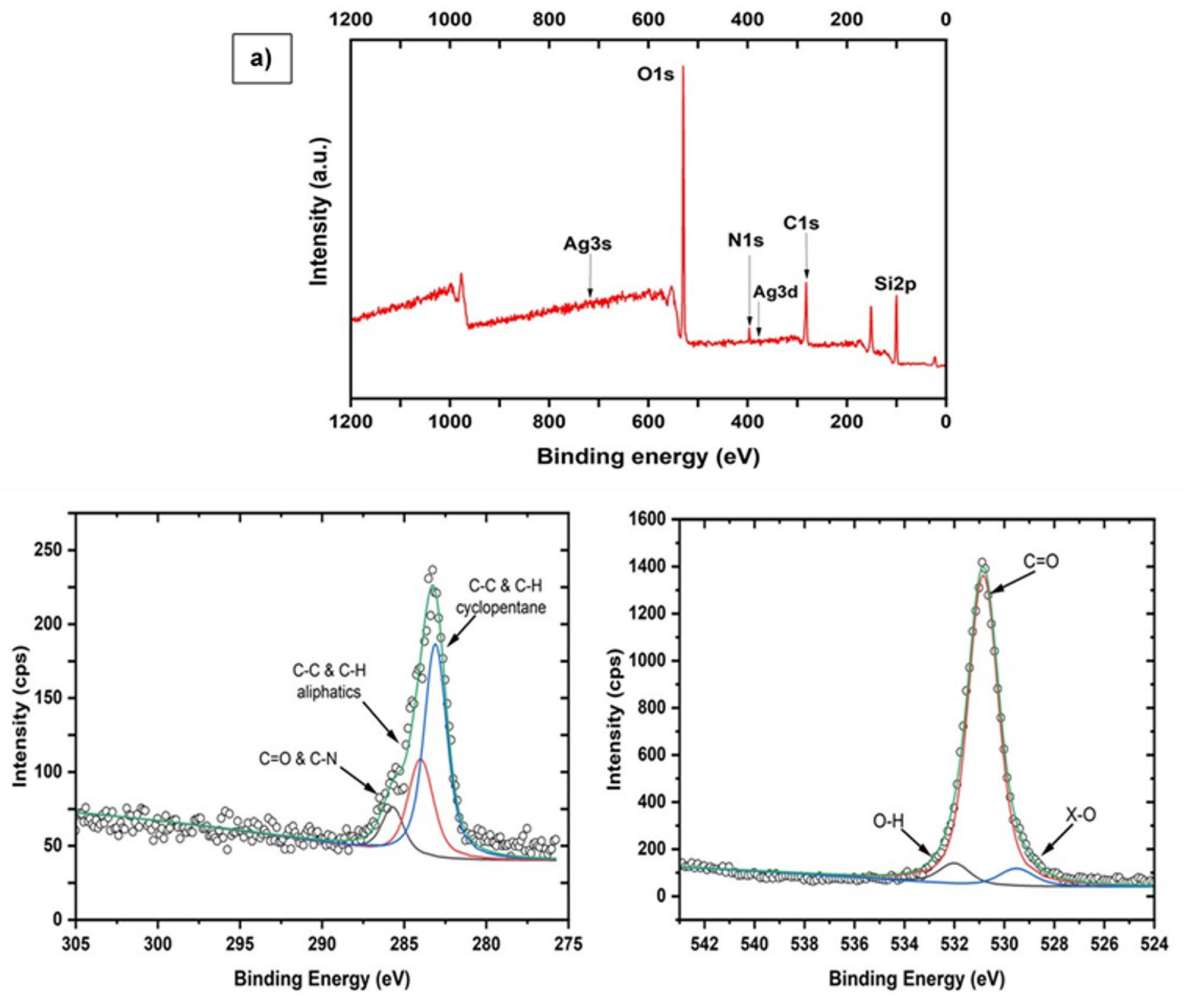
(**a**) Complete XPS PVP-AgNPs spectrum. Observed peaks indicate the presence of carbon (C), nitrogen (N), oxygen (O), silicon (Si) and silver (Ag) in different electronic configurations in the nanocomposite sample. (**b**) High-resolution XPS spectra deconvolution of C1s. Three peaks associated with bonds formed by carbon with other elements are observed. (**c**) High-resolution XPS spectra deconvolution of O1s. Three peaks are observed that correspond to the bonds that oxygen forms with other elements.

To determine the elemental composition of the nanocomposite in a qualitative and quantitatively way, an XPS analysis was performed. As shown in Fig. 3a, the complete XPS spectrum obtained shows the presence of silver, oxygen, nitrogen, carbon, and silicon traces. When comparing the XPS spectrum reported in previous investigation^37,38^, with our results, it was noticed that silver peaks showed greater intensity in previous research than that displayed by our obtained data. This fact could be explained due to different factors, such as the equipment sensitivity, the measurement angle, or the different silver oxidation states at the measurement moment.

The XPS equipment used in this research has a penetration power of only 100 nm so, it is possible that, due to the polymer coating width, the silver signal was not completely captured explaining the poor penetration, leading to a silver low intensity. Table 1 presents the complete elemental PVP-AgNPs composition percentage (%) obtained from the XPS spectrum. As part of the XPS analysis, the high-resolution carbon and oxygen spectrum, showed small variations in both, carbon and oxygen binding energy due to the environments in which they were found during the measurement.

**Table 1:** Complete elemental PVP-AgNPs composition percentage (wt%) obtained from the XPS spectrum.

Element	Composition (wt%)
Carbon	18.11
Oxygen	50.37
Sodium	0
Silica	25.99
Chlorine	0.57
Silver 3d	0.11
Silver 4p	0
Silver 3s	1.66

This spectrum was deconvolved into the elements components peaks and their positions in terms of binding energy and, in such a way, to determine the possible interactions between both components, carbon and oxygen, in the environment in which the PVP-AgNPs were found during measurement^39^.

Deconvolution of C1s depicted in Fig. 3b shows three peaks. Two of them, in the C-C binding region and other one in the oxygenated region. The first one as shown in the figure, is located in the oxygenated region, at a binding energy corresponding to 285.7 eV indicating the presence of carbonyl groups or carbon – nitrogen (C-N) bonds^40,41^.

The other two are located in the C-C region. One of them, is located at the binding energy of 284 eV, which corresponds to C-C and C-H bonds of aliphatic compounds and the last peak is found at the binding energy of 283.105 eV, which corresponds to cyclopentane bonds. Generally, this peak is located at a binding energy of 283.4 eV^41^.

Figure 3c shows O1s deconvolution presenting 3 peaks. The first is located at a binding energy of 532 eV, which corresponds to ether or hydroxyl groups attached to aliphatic compounds; this peak is usually found between 532.5 and 532.9 eV. The second peak is found at a binding energy of 530.85 eV which corresponds to carbonyl, lactones or carboxyl groups, which is consistent with the type of bond shown in the deconvolution of C1s. The common location of this specific peak is between 531.3 and 532.6 eV^41^. The last peak is found at a binding energy of 529.52 eV which corresponds to metal oxides that is within the reported range of 529–530 eV^42^.

The little binding energies shifts found in the deconvolved peaks, can be influenced by factors such as nanoparticles sizes, interactions with the medium, deformations in the material crystalline lattice or the material relaxation effects in the final state of the sample after X-rays excitation^43^.

In this case, the interaction between the positive partial charges of the AgNPs and the negative partial charges of the PVP carbonyl groups, joined to both, the complex formation between the carbonyl groups or the PVP nitrogen ring with silver^44^ and the nanoparticles size, can explain the slight displacements observed in our results.

### PVP-AgNPs stability over time

**Fig. 4:**
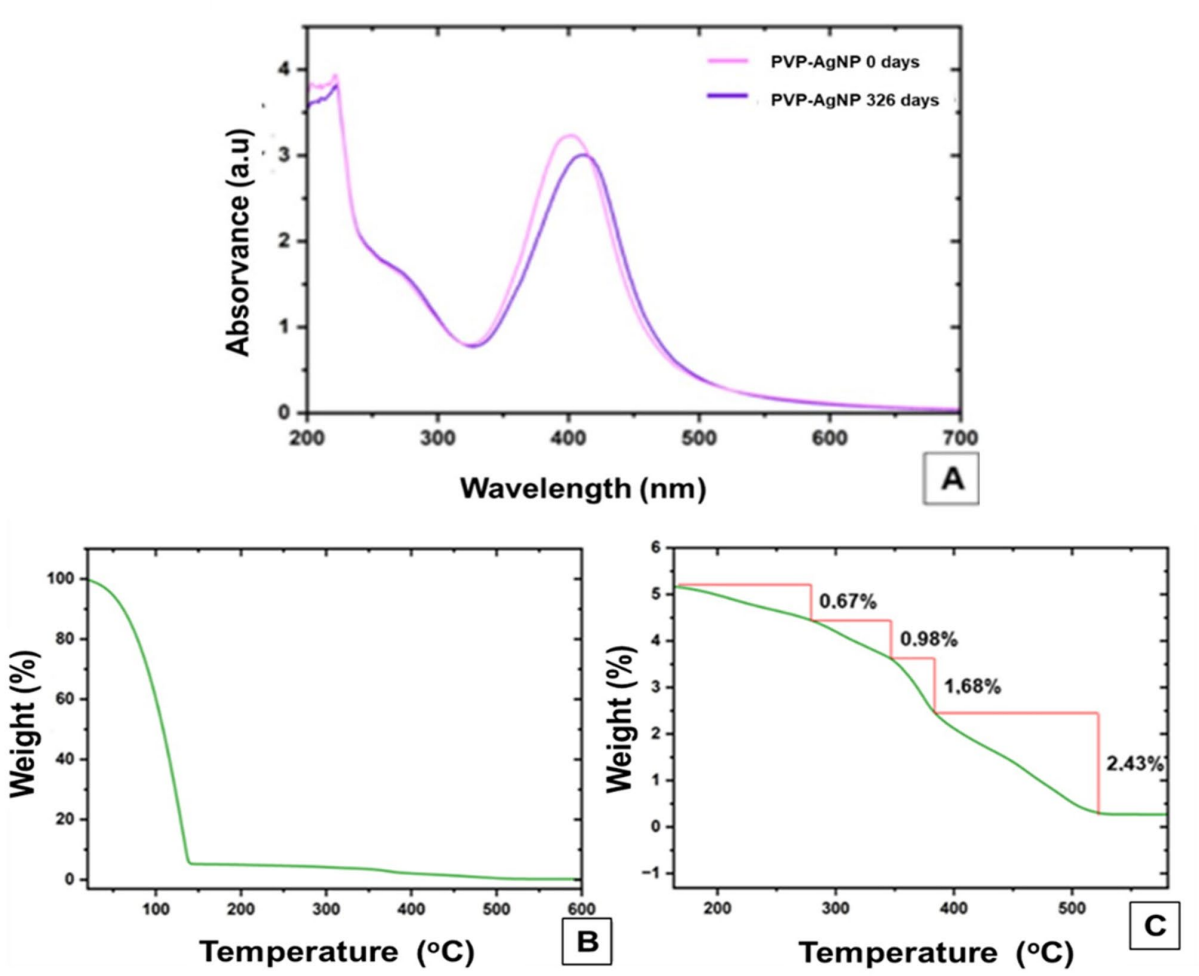
(**a**) PVP-AgNPs UV–Vis spectrum from day 0 (Pink line) and after 326 days (blue line). (**b**,**c**) TGA analysis of PVP-AgNPs stability under an oxidizing atmosphere. (**b**) Mass loss due to water evaporation in the range of 16 °C to 140 °C. (**c**) Close-up of the interval between 200 and 500 °C showing the loss of mass due to degradation of PVP-AgNPs.

The final objective of the PVP-AgNPS nanocomposite is to control pests in organic agriculture so, real-time stability is an important parameter to be evaluated. Figure 4a shows the stability curve of PVP-AgNPs during 326 days at 25–30ºC. During this period, absorbance values decreased on just only 0.2295 units, going from 3.2371 to 3.0076 units. Furthermore, a slight shift of the absorbance peak was recorded, from 401 nm to 414 nm.

These small changes in absorbance over the long period of time tested indicated that the PVP-AgNPs are highly stable, retaining their integrity and concentration even for long periods of time under room temperature. A similar behavior was reported by other investigations^45^, where also the stability of silver nanoparticles coated with PVP was evaluated but only for 120 days (4 months), without observing a significant decrease in absorbance. However, our study was conducted in a longer period of time with no-refrigeration at room temperature conditions. This fact is really very important because this condition is closer to that in which PVP-AgNPs will be used.

Small variations in wavelength observed in Fig. 4a could be related to small changes in the optical properties of the dissolved organic matter^46^. In this case, given that the PVP-AgNPs were synthesized using green chemistry, it is likely that they contain residues of organic matter from the rosemary extract, which could explain the observed shift in wavelength, which does not affect the integrity of PVP-AgNPs nanocomposite. The nanocomposite thermal stability was also corroborated through a thermogravimetric (TGA) analysis. Figure 4b shows the stability thermogram, where a large descent curve is observed that reflects the mass loss of 94.24% between a temperature range of 21 to 142 °C. A thermogram close-up was made as depicted in Fig. 4c to show decreases in the stability curve between a range of 150 to 650 °C. Four important variations occurred as judged by this figure which represented the organic compounds degradation present in the sample. Firstly, a mass loss of 0.67% between 151 and 266.5 °C was observed. Afterwards, another decrease in the curve was found at a range of 266.5 and 350 °C corresponding to 0.98%. On the other hand, a degradation interval represented a mass percentage of 1.68% between 350 and.

393.2 °C, and finally the steepest curve at a temperature of 400 to 520 °C showed a percentage of 2.43%.

These results agree with previous research^47,48^ where the presence of the PVP polymer in the AgNPs nanocomposite was thermally analyzed. In this cited research, a first weight loss up to 140 °C was described due to the evaporation of the adsorbed water and the humidity contained in to the PVP-AgNPs medium. In the second descent of the curve to 251 °C they observed the loss of the residual solvent and from the third curve descent, the decomposition of PVP that is found around the AgNPs from 250 to 500 °C. Interesting, it should be emphasized that in their research the molecular weight of the PVP used was 40,000, unlike that used in this research, with a molecular weight of 10,000. The mass loss of PVP between 250 and 500 °C depends on the molecular weight used in the nanocomposite synthesis^47,48^.

Contrary to our findings, other investigations reported that AgNPs coated with PVP but synthesized by chemical methods were not stable^49^. In these investigations, silver nitrate (AgNO_3_) was reduced with sodium borohydride (NaBH_4_), using PVP with two different molecular weights (40 000 k and 55 000 k) as stabilizing agents.

### Insecticidal activity of PVP-AgNPs

**Fig. 5:**
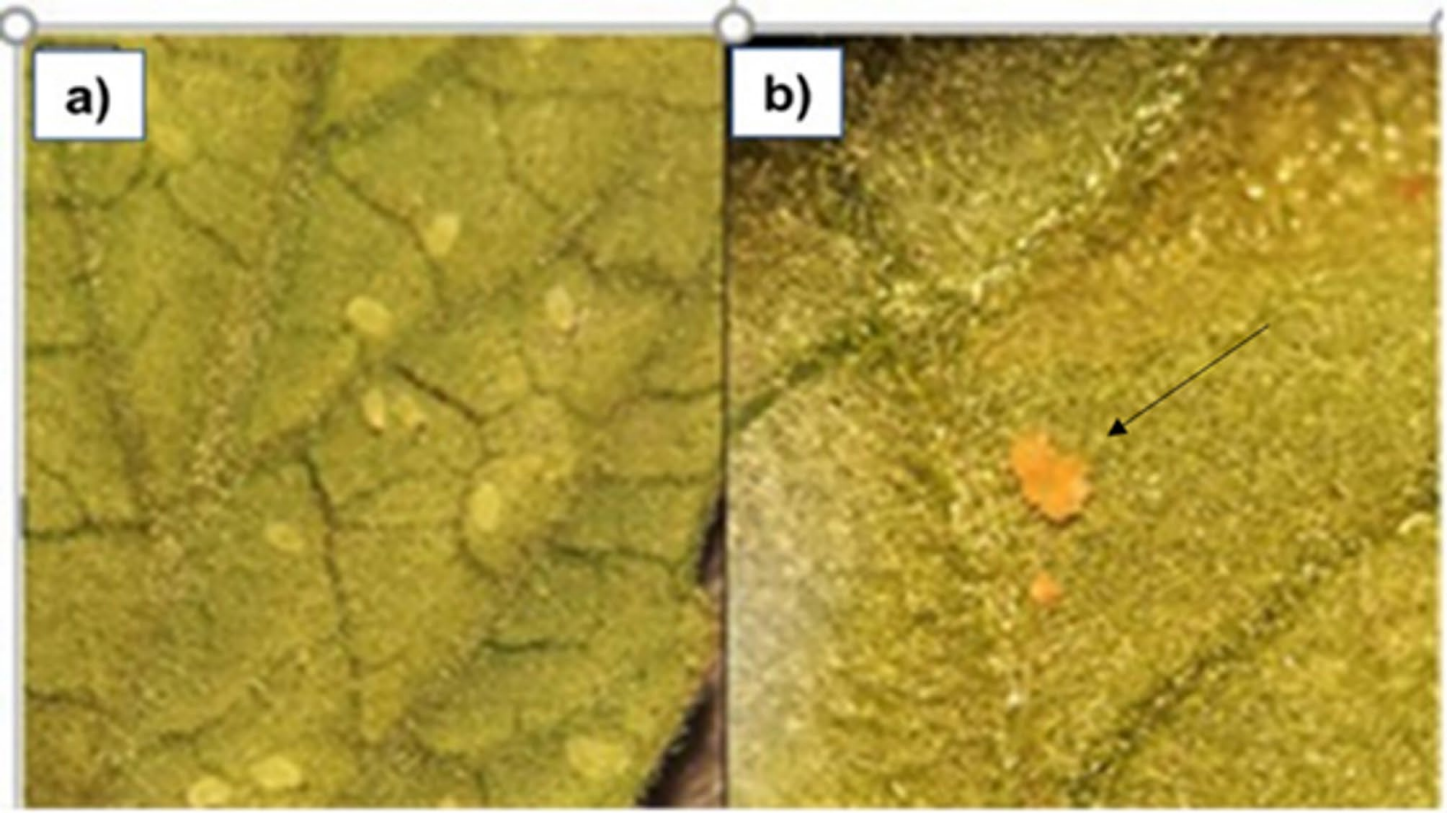
Insecticidal effect of AgNPs–PVP. (**a**) Presence of live whitefly individuals on tomato leaves. (**b**) Signaled with a black arrow, a magnified picture indicating the presence of dead individuals after 10 days of the nanocomposite application with a change in color from transparent to brown indicative of mortality.

Table 2 shows that 10 days (240 h) after the spraying the nanocomposite on infected leaves, a mortality percentage of different biological stages of the white fly was 98.0, 97.84 and 96.1 for concentrations of 64 ppm, 32 ppm and 16 ppm respectively, while a lower mortality rate was observed for the rest of the assayed dilutions. After 10 days, an affectation is also seen in eggs, which changed from transparent Fig. 5a to a dark brown color as shown in Fig. 5b. Also, Table 2 display that already at 24 and 48 h after spraying both, the nanocomposite and its dilutions there was an important lethal effect on the larvae and nymphs present in the leaves mainly in the two highest concentrations of 64 and 32 ppm.

**Table 2:** Mortality % of different individuals of whitefly after 24, 48 and 240 h (10 days), after spraying the leaves with the 64 Ppm nanocomposite solution and dilutions 32, 16, 8, 4 Ppm respectively.

PVP-AgNPs concentrations	24 h	48 h	240 h
64 ppm	47.20 ± 2.4a	90.66 ± 1.65a	98.0 ± 0.00a
32 ppm	45.83 ± 1.26a	87.40 ± 1.08a	97.84 ± 0.76a
16 ppm	34.87 ± 1.56b	82.45 ± 1.35b	96.10 ± 1.95a
8 ppm	12.30 ± 0.5c	35.3 ± 1.25c	44.80 ± 1.2b
4 ppm	1.10 ± 0.56d	16.40 ± 1.25d	22.5 ± 1.2c
LC^50^	57.7	10.4	7.6

The percentage data were transformed by arcsine square root for further processing. Tabulated data correspond to the means of 4 replicates ± one standard deviation. Means with the same letter are not statistically different (Tukey *p* < 0.05).

To date, exist very few reports related to the action of silver nanocomposites against different stages of the greenhouse whitefly so, this work could be a reference for subsequent studies of the PVP-AgNPs mechanisms of action against this important pest that causes great losses, especially in organic crops where chemical pesticides are not used for its control.

Green PVP-AgNPs synthesis, using plants extracts as reducing and trapping agents, has shown to cause mortality in almost all stages of mosquitoes, that is, egg, larva and adult, in addition to producing acute toxicity, fumigant, antifeedant, repellent and attractant properties, acting as reproductive inhibitors for many species of agricultural and stored product pests, at a lower dose than chemically synthesized nanoparticles^50^.

However, precise information on the mechanisms of nanoparticle action against insects and mites is still very limited. Silver nanoparticles have been shown to have a significant impact on antioxidant and detoxifying enzymes in insects, leading to oxidative stress and cell death^51^. Silver nanoparticles also reduced acetylcholinesterase activity in some insects. Metallic nanoparticles in general can bind to proteins and nucleic acids, respectively, leading to a decrease in membrane permeability and thus denaturation of organelles and enzymes, followed by cell death.

Furthermore, Ag nanoparticles up- and down-regulate key insect genes, reducing protein synthesis and gonadotropin release, leading to developmental damage and reproductive failure. The insecticidal properties of AgNPs can be attributed mainly to their morphology, dimensions and high covering power, which favors their penetration into the insect body^52^. One of the advantages of using AgNP as a control agent is its low risk of developing resistance in prolonged use^53^.

### Antimicrobial activity of PVP-AgNPs nanocomposite

**Fig. 6:**
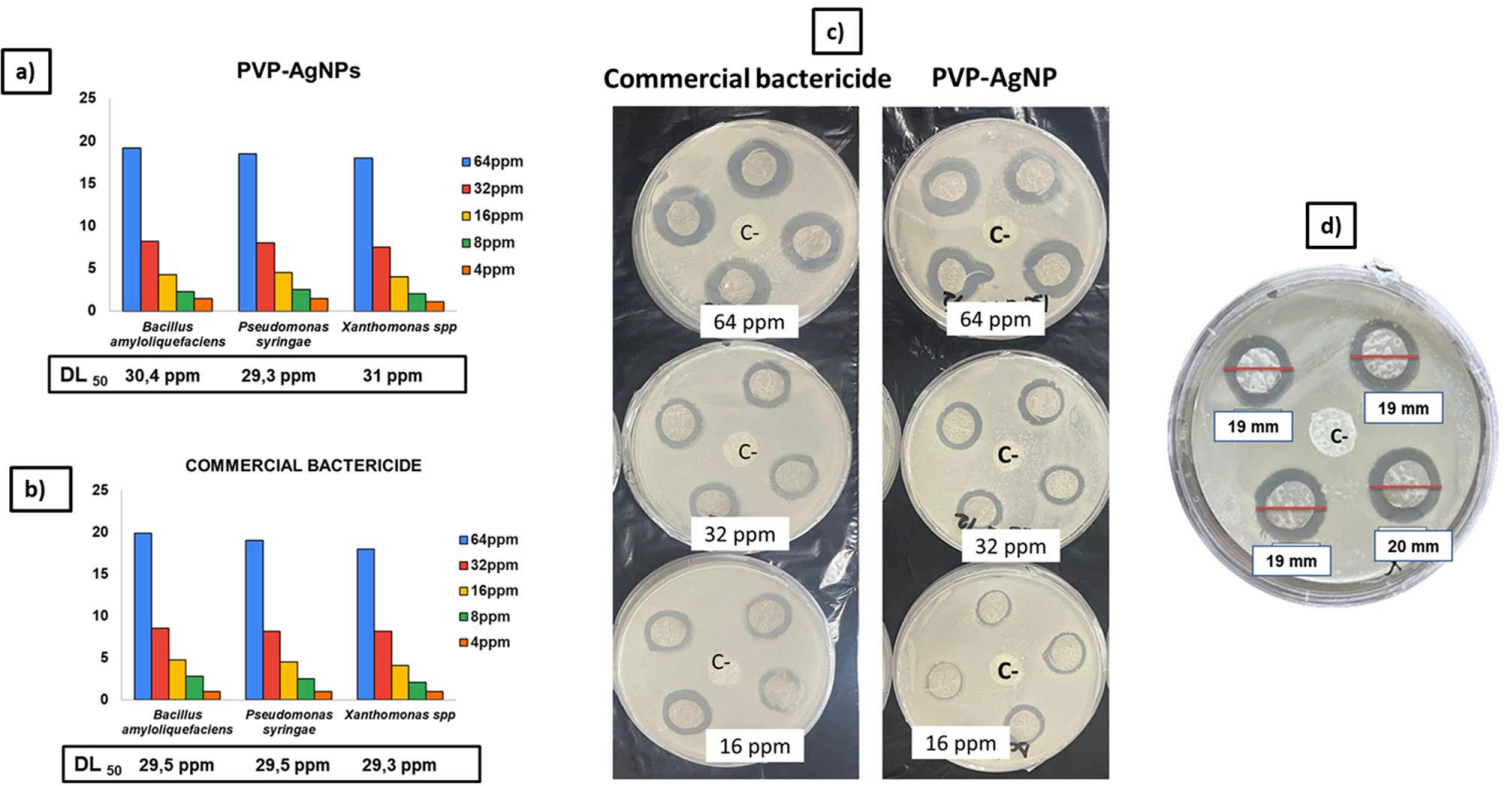
(**a**,**b**) Growth inhibition of 3 bacterial isolates corresponding to *Bacillus amyloliquefaciens*,* Pseudomonas syringae and Xanthomonas* spp after the application of 64 ppm of the nanocomposite or a commercial pesticide and its dilutions corresponding to 32,16,8 and 4 ppm respectively. The graphed data showing also DL_50_ and IC_50_ values, correspond to the average values of the 4 replicates present in each Petri dish ± one standard deviation (SD). The inhibitory activity is represented by the diameter of the growth inhibition halo (mm) including disc area as shown in the illustrative figure (**c**,**d**) corresponding to the experiment carried out using only the bacterium *B. amyloliquefaciens* and how the halo measurement was carried out. Commercial pesticide was used in parallel as control in all these experiments.

Figure 6 shows the results of the agar disk diffusion method as described in methods to evaluate the inhibitory effect of the nanocomposite and its dilutions on the growth of 3 bacteria, *Bacillus amyloliquefaciens* (non-pathogenic bacteria), *Pseudomonas syringae* causing agent of bacterial spot and *Xanthomonas* spp causing bacterial spot of tomato. The bactericidal effect over these three bacterial species was also studied in parallel with a commercial bactericide.

Inhibition zone values measured in mm of diameters presented in Fig. 6a–c after applying 80 *µ*L of the 64 ppm solution of the nanocomposite or the commercial pesticide in the corresponding plates and its dilutions corresponding to 32, 16, 8 and 4 ppm respectively for 48 h.

In all cases there was a growth inhibition which was more pronounced in the undiluted variant of the nanocomposite corresponding to 64 ppm, although inhibitory effects were also observed in the rest of the variants. The exact inhibition mechanisms of AgNPs against bacteria still remain unknown. However, there are some researchers who propose that the action of AgNPs on bacteria may be due to their ability to penetrate the cell^54^, the formation of free radicals^55,56^, the inactivation of proteins in the cell by silver ions and the production of reactive oxygen species (ROS)^57^. In addition, there are also some other factors that influence the bactericidal action of AgNPs, such as AgNPs, concentration, the.

bacteria type^58^, the shape and AgNPs size^59^.

**Fig. 7:**
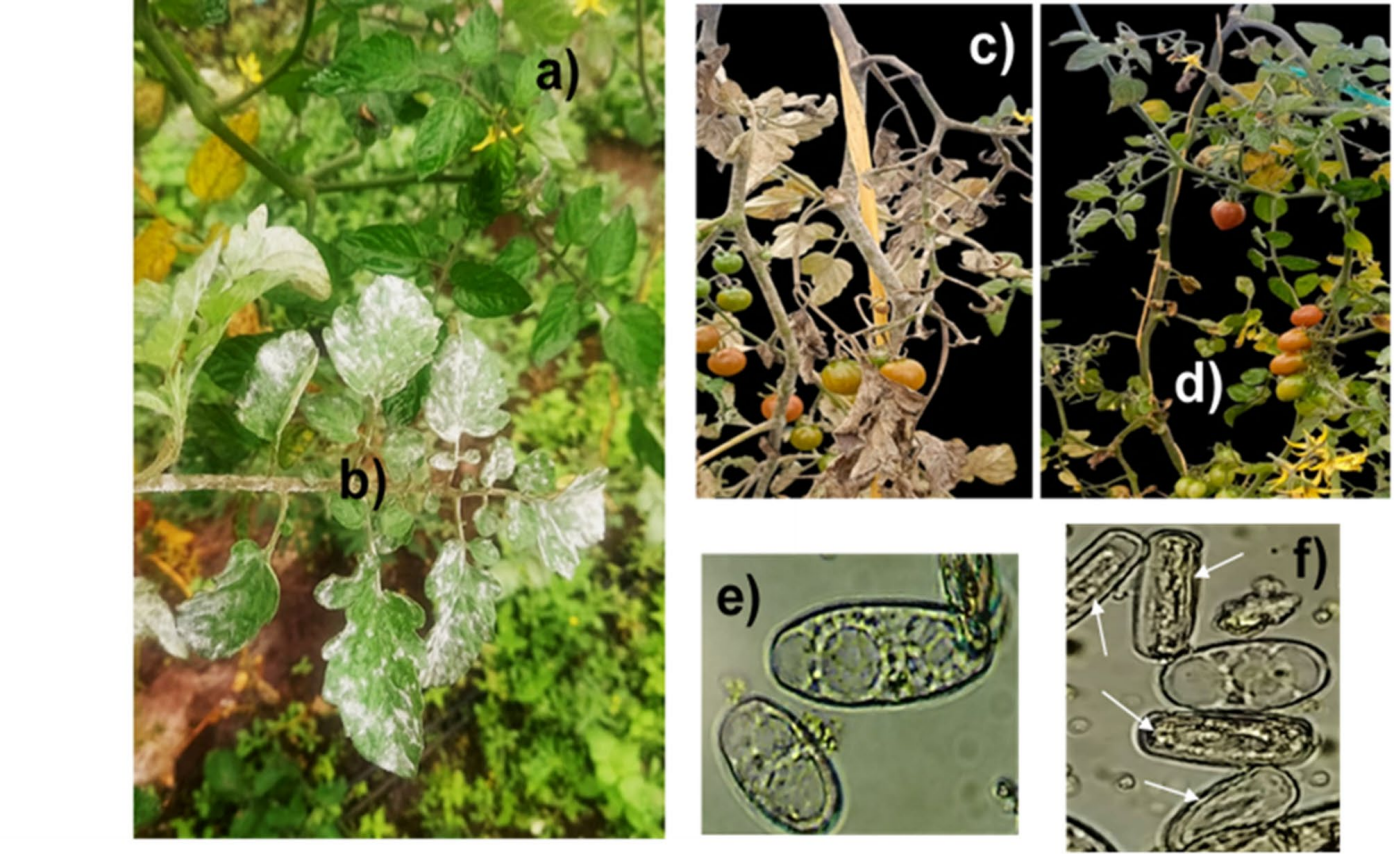
Greenhouse grown plants naturally infected with *Oidium neolycopersici*: (**a**,** d)** Sprayed plants with the PVP-AgNPs formulation displaying no symptoms and no apparent fungi infection, (**b**,** c)** Nontreated plants showing severe fungal attack and disease symptoms. Light microscopy showing (**e**) normal fungal cells from individualized tomato leaves treated with water and (**f**) fungal cells from individualized infected leaves treated with the PVP-AgNPs solution showing damaged cells with white arrows.

Regarding *Oidium neolycopersici* control, Fig. 7b, c show greenhouse grown tomato plants that were not sprayed with the 64 ppm PVP-AgNps nanocomposite solution. These non-treated plants as shown in the figures were severely affected by the fungi. The most characteristic symptoms of powdery mildew in tomato correspond to whitish, powdery spots on the leaves upper part, which later turn yellowish becoming necrotic. If the disease progresses, it causes plant defoliation and arrest of its growth. Conversely, no disease symptoms were observed in the neighboring sprayed plant as judged by Fig. 7a, d, indicating PVP-AgNPS effectiveness against this powdery mildew. Light microscopy confirmed the detrimental effects of PVP-AgNps on fungal cells hyphae, germination and cellular disruption as shown in Fig. 7f when compared with non-treated, no damaged fungal cells (Fig. 7e).

In previous research, the antifungal activities of the AgNPs synthesized using green or chemical methods have been studied^60^. For both cases, the synthesized AgNPs effectiveness depends rather on the used dose and the target fungus. Regarding toxicity and minimum inhibitory concentrations, differences were also reported. The chemically synthesized AgNPs were more toxic against the target organism and their toxicity was dose dependent. However, the minimum inhibitory concentrations of green-synthesized AgNPs were compared to those of chemically synthesized AgNPs indicating higher antifungal potency of green AgNPs than that chemically synthesized^61^. This fact could justify the results we show in this research regarding by the first time green sinthesized PVP-AgNPs effectiveness against *Oidium neolycopersici*.

On the other hand, it has been observed that plant extracts used for green AgNPs synthesis is also an important factor in terms of effectiveness to control certain types of fungi^62^. Leaves or roots of different plants extracts used as reducing agents in green AgNPs synthesis such as *Hyllanthus urinaria*,* Pouzolzia zeylanica*,* Scoparia dulcis*,* Acalphya indica*,* Aloe vera*,* Brassica oleracea*,* Cassia roxburghii*,* Diospyros sylvatica*,* Diospyros paniculata*, have proven, in one way or another, to produce AgNPs with some effectiveness against different fungi species such as *Aspergillus niger*,* A. flavus*,* Fusarium oxysporum*,* Alternaria alternata*,* Botrytis cinerea*,* Curvularia lunata*,* Macrophomina phaseolina*,* Rhizoctonia solani*,* Sclerotinia sclerotiorum*^63–66^. To date, no reports exist on the use of rosemary extracts to get green synthesized PVP-AgNPs able to control *Oidium neolycopersici* on greenhouses grown tomato plants.

Similar to the insecticide and bactericide effect, the exact fungicide PVP-AgNPs mechanisms and modes of action need to be further explored. However, the antifungal activity of nanoparticles is attributed to its smaller size and large surface ratio. AgNPs toxicity to fungal cells is partially attributed to their production of reactive oxygen species (ROS), leading to apoptosis. Other fungal cell damages caused by nanoparticles toxicity includes: disintegration and deformation of the cell wall and cell shape, proteins and nucleic acids damage due to production and accumulation of ROS and free radicals, blockage of proton pumps, blockage of cellular respiration and, therefore, damage to the electron transport system^67,68^.

## Methods

### Materials

For PVP-AgNPs nanocomposites preparation, the biodegradable polymer polyvinylpyrrolidone (PVP) 4000 (Sigma-Aldrich), sodium hydroxide (Sigma - Aldrich), silver nitrate AgNO_3_ (Fisher Scientific) were used. Plants for rosemary extract, were purchased in a supermarket (Quito, Ecuador) and 96% ethanol was purchased in the Casa del Químico, Quito, Ecuador. All solutions used in this research were prepared with type I distilled water.

### Obtaining Rosemary extract (*Rosmarinus officinalis* L.) used as silver reducing agent

The procedure was carried out according previously report^69^, but with our own modifications. 50 g of rosemary leaves were weighed, washed three times with current water and twice with distilled water. Subsequently, leaves were placed in a 100 mL glass bottle containing a solution composed of 75 mL of 96% ethanol and 25 mL of distilled water. The jar was then covered with an aluminum foil and allowed to sit for 4 days at room temperature in a dark place. After 4 days, a dark green solution was obtained, which was strained and transferred to a balloon, which was placed in a Bucci II rotary evaporator. The evaporation process was carried out at 40 °C with a water bath and at a pressure of 85 mbar for 3 h, to extract all the ethanol. From the concentrated extract obtained, 20 mL aliquots were taken, which were diluted with distilled water up to 100 mL. These dilutions were subjected to vacuum filtration using a 0.45 *µ*m pore size membrane. Once filtered, the solutions were placed in 50 mL Falcon tubes and stored at 4 °C.

### Synthesis of PVP-AgNPs nanocomposite

This scalable procedure was carried out in a general way following previously reported protocols^70^, with our own modifications that allowed us to obtain the nanocomposite in a single step. 120 mL of distilled water was heated in a Termoline stirring plate until reaching 90 °C and then 1 g PVP was added. It was stirred at 2000 rpm until the polymer was completely dissolved and 10 mL of 0.1 M sodium hydroxide and 20 mL of 0.01 M silver nitrate were immediately added. After 5 min, 16 mL of rosemary extract (*Rosmarinus officinalis* L.) was added and allowed to react for 3 h. All the characterization tests of the obtained nanocomposites presented below were carried out in the advanced materials laboratory of the ESPE Armed Forces University and the Yachay Tech University, Ecuador.

### UV–Vis spectrophotometer characterization of the PVP-AgNPs nanocomposite

A Thermo Scientific Genesys 10 UV spectrophotometer was used. In a 4 mL capacity quartz cell, 3 mL of type 1 distilled water was placed to generate the baseline and blank. Once the baseline was obtained, the water contained in the cell was discarded and 3.5 mL of type 1 distilled water was added. together with 100 *µ*L of the obtained PVP/AgNPs nanocomposites or just only AgNPs for the case of the control group. Both, PVP/AgNPs nanocomposites or AgNPs were obtained after three hours reaction. The quartz cell was then covered and homogenized by immersion, placed in the equipment and run with scanning conditions at wavelengths from 200 to 750 nm.

### PVP-AgNPs nanocomposite characterization in a particle analyzer

The characterization of the PVP-AgNPs was carried out in a Horiba In Color Dynamic Light Scattering Particle Size Analyzer LB – 550 brand particle analyzer. The characterization conditions in terms of refractive index were values of 0.190–3.400i for the AgNPs and for the dispersant, in this case water, 1.333 with a density of 1 g/cm^3^. To carry out the measurement, 3.5 mL of type 1 distilled water was placed in a 4 mL capacity quartz cell, to which 10 *µ*L of PVP-AgNPs obtained after 3 hours reaction were added. For the control group, instead of PVP-AgNPs, the uncoated AgNPs were used. The quartz cell was then covered and homogenized by immersion, and subsequently placed in the equipment. The temperature control was carried out at 25 °C by the equipment internal electrode.

### PVP-AgNPs nanocomposite characterization by X-ray photoelectron spectroscopy (XPS)

Drops of the PVP-AgNPs nanocomposite were placed on a sample dake for previous drying. Then, the dake was placed in the XPS chamber (Alter technology SA.), closed until ultra-vacuum conditions were reached. Subsequently, the measurement protocol was followed using the program recommended by the manufacturers.

### Thermogravimetric analysis of the PVP-AgNPs nanocomposite

For thermogravimetric analysis of the nanocomposite, a TGA 55 equipment (Waters.SA) was used. The sample carrier crucible was weighed and a sample drop was subsequently placed and entering directly into the equipment oven. The equipment was programmed with a temperature range from 0 to 650 °C, speed of 10 °C per minute and oxidizing atmosphere. The test was run for one hour and thirty minutes.

### Insecticidal activity of PVP-AgNPs against *Trialeurodes vaporariorum*

PVP-AgNPs was applied in sprayed doses of 64, 34, 16, 8, 4 ppm respectively, to naturally infested tomato leaves taken from infested greenhouse grown tomato plants showing different biological stages of the whitefly. Six damaged leaves were cut at random from different plants per replicate, including the control so, for 3 replicates, 18 infested leaves that had different stages of the fly were used for each PVP-AgNPs dose. Sixty insects per leaf, along with their replicates, were analyzed in each experiment after samples homogenization under a stereo-microscope taking into account the number of individuals. The physiological status of the insect’s life cycle present in the leaves samples was primarily determined by the egg stage and first and second instar larvae.

Each individualized infested leaf was placed in a 30 × 10 cm plastic container with a lid. Before applying the nanocomposite, individuals were previously counted on the leaf underside with the help of a stereomicroscope. Afterwards, absorbent paper was placed and distilled water was sprayed to keep the leaves hydrated. Subsequently, the nanocomposite was applied depending on the dose (64, 32 or 16 ppm) directly to the leaves with a 50 mL capacity atomizer, covering the entire leaf, without leaving unwetted areas. The 6 leaves corresponding to the control were sprayed with only distilled water. After 24, 48 h to 10 days after the application, the counting of individuals was carried out again, but this time distinguishing between alive or dead individuals compared with the water treated controls. Nymphs’ mortality or eggs hatching in treated leaves were used as mortality criterion and compared with control leaves infested only treated with water.

### Bactericidal activity of the PVP-AgNPs nanocomposite

The bacterial growth inhibition test was carried out by the agar disk diffusion method using 100 *µ*L of a standardized inoculum suspension of each bacterium to be evaluated containing 10^7^ CFU/ml of each one^71^. For this purpose, a bacterial lawn was obtained from 3 individual bacterial strains, *Bacillus amyloliquefaciens*,* Pseudomonas syringae and Xanthomonas* sp, isolated directly from tomato and babaco crops respectively. These 3 strains were previously microbiologically and molecularly characterized to confirm their identity and purity. On the grass of each Petri Agar LB plate were placed filter paper discs obtained from a 1.3 mm punch, that were previously sterilized for 1 h at 120 °C and 15 psi pressure. Five paper discs were placed on each plate and 80 µL of sterile distilled water was placed on the disk located in the center of the plate and 80 *µ*L of the nanocomposite was placed on the remaining 4 disks depending on the dilution to be used (64, 32, 16, 8, and 4 ppm). The plates were sealed with Parafilm and incubated at 25 °C for four days. After this time, the bacterial growth inhibition zones were measured.

### Plant materials

Commercial tomato varieties used in this work were Fortuna and Stella varieties, both Ecuadorian varieties obtained from MEGAPLANTS coorporation https://www.megaplantec.com/disponibilidad-de-plantas/ and ECUA- PLANTAS coorporation https://ecuaplantas.com/. 10 tomato plants were grown under controlled greenhouse conditions (28 °C, 70% relative humidity, approximately 2,000 lx with a 12 h natural light/12 h darkness photoperiod). After four weeks 5 of them were sprayed with the 64 ppm PVP-AgNPs nanocomposite solution. Plant damages occurring by naturally oidium infection were followed during 1 month.

### Determination of *Oidium neolycopersici* cells damage after PVP-AgNPs treatment

Leaves with a high degree of oidium infection obtained from non-PVP-AgNPs treated plants, covering the entire leaf area were individualized in plastic containers measuring 10 by 20 centimeters. For three replicates of the PVP-AgNPs treatment, 3 leaves were taken to get 9 leaves for each 3 replicates. Additionally, nine infested leaves were used for the 3 replicates of the untreated control. Before applying the product, the physiological state of the fungal spores was observed for both, PVP-AgNPs and control treatments. Subsequently, the 64 ppm solution of the product was sprayed on all the replicas corresponding to the PVP-AgNps treatment and in the case of the control, sterile distilled water was sprayed. The state of the cells was observed under a light microscope after 24 h after de treatment.

### Light microscopic observation

Leaves naturally infected with Powdery mildew were chosen from plants grown under greenhouse conditions. Scotch tape was used to collect the spores to be able to observe unaffected and affected spores after de PVP-AgNPs treatment. Then, spores’ morphological traits were observed under a light microscope equipped with software (OPTIKA PROVIEW, Italy).

### Statistical analysis

Data analysis was performed in SAS OnDemand for Academics software (online version). All tabulated or graphed data were expressed as the mean ± SD (standard deviation) of three independent experiments. When necessary, to determine differences in mortality between treatments, the data were transformed using the arcsine/square root. Statistical significance between the treated and control groups was analyzed by Tukey’s test using GraphPad software version 9.1.1. (San Diego, California, USA). The value of *p* < 0.05 was considered statistically significant to consider the differences.

## Data Availability

Datasets used and/or analysed during the current study are available from the corresponding author on reasonable request.
