# Publisher Correction: Pesticide effects of highly stable green synthesized silver nanocomposites to be used in organic tomato crops

**DOI:** 10.1038/s41598-025-16467-7

**Published:** 2025-08-20

**Authors:** Luis E. Trujillo, Pablo Landázuri, Carlos Noceda, José Velasco, Luz Toapanta, Giulliana Criollo, Andrea Poaquiza, Fulton Barros, Vladimir Aguirre, Andrés Izquierdo, Julio Chacón, Zailmar Morales, Alexis Debut

**Affiliations:** 1 Life Science and Agriculture, Universidad de Las Fuerzas Armadas-ESPE, Industrial Biotechnology Research Group Sangolquı, Sangolquí, 171103 Ecuador; 2 Center for Nanoscience and Nanotechnology (CENCINAT), Universidad de Las Fuerzas Armadas (ESPE), Sangolquí, 171103 Ecuador; 3 Yachay Tech.University, Urcuquí, 100115 Ecuador

Correction to: *Scientific Reports* 10.1038/s41598-025-03101-9, published online 01 July 2025

The original version of this Article contained a technical error in the author list.

The original Article has been corrected.

